# Bis-pharmacophore of cinnamaldehyde-clubbed thiosemicarbazones as potent carbonic anhydrase-II inhibitors

**DOI:** 10.1038/s41598-022-19975-y

**Published:** 2022-09-27

**Authors:** Asif Rasool, Zahra Batool, Majid Khan, Sobia Ahsan Halim, Zahid Shafiq, Ahmed Temirak, Mohamed A. Salem, Tarik E. Ali, Ajmal Khan, Ahmed Al-Harrasi

**Affiliations:** 1 Institute of Chemical Sciences, Bahauddin Zakariya University, Multan, 60800 Pakistan; 2 Natural and Medical Sciences Research Center, University of Nizwa, Nizwa, Sultanate of Oman; 3 Department of Pharmaceutical and Medicinal Chemistry, University of Bonn, An der Immenburg 4, 53121 Bonn, Germany; 4 National Research Centre, Chemistry of Natural and Microbial Products Department, Pharmaceutical and Drug Industries Research Institute, Dokki, P.O. Box 12622, Cairo, Egypt; 5 Department of Chemistry, Faculty of Science and Arts, King Khalid University, Muhayil, Assir Saudi Arabia; 6 Department of Chemistry, Faculty of Science, Al-Azhar University, 11284 Nasr City, Cairo, Egypt; 7 Department of Chemistry, Faculty of Science, King Khalid University, Abha, Saudi Arabia; 8 Department of Chemistry, Faculty of Education, Ain Shams University, Cairo, Egypt

**Keywords:** Enzymes, Organic chemistry, Medicinal chemistry, Structure-based drug design

## Abstract

Here, we report the synthesis, carbonic anhydrase-II (CA-II) inhibition and structure–activity relationship studies of cinnamaldehyde-clubbed thiosemicarbazones derivatives. The derivatives showed potent activities in the range of 10.3 ± 0.62–46.6 ± 0.62 µM. Among all the synthesized derivatives, compound **3n** (IC_50_ = 10.3 ± 0.62 µM), **3g** (IC_50_ = 12.1 ± 1.01 µM), and **3h** (IC_50_ = 13.4 ± 0.52 µM) showed higher inhibitory activity as compared to the standard inhibitor, acetazolamide. Furthermore, molecular docking of all the active compounds was carried out to predict their behavior of molecular binding. The docking results indicate that the most active hit (**3n**) specifically mediate ionic interaction with the Zn ion in the active site of CA-II. Furthermore, the The199 and Thr200 support the binding of thiosemicarbazide moiety of **3n**, while Gln 92 supports the interactions of all the compounds by hydrogen bonding. In addition to Gln92, few other residues including Asn62, Asn67, The199, and Thr200 play important role in the stabilization of these molecules in the active site by specifically providing H-bonds to the thiosemicarbazide moiety of compounds. The docking score of active hits are found in range of − 6.75 to − 4.42 kcal/mol, which indicates that the computational prediction correlates well with the in vitro results.

## Introduction

Carbonic anhydrases are broadly distributed metalloenzymes in both eukaryotes and prokaryotes. They efficiently catalyze the reversible hydration of CO_2_ to HCO_3_^−^ and H^+^ ions and perform a significant function in the regulation of many pathological and physiological processes^1–5^. As a result, these enzymes are arousing targets for the treatment of pathological diseases^6–8^. CA-II is greatly associated with maintaining the concentration of bicarbonate in the eyes^9–12^. These inhibitors have been examined as a supplement in cancer chemotherapy^12^. Besides, CA-II is also reported in renal, pancreatic, and gastritis carcinomas and malignant brain tumors^13–15^. Carbonic anhydrase activity from bovine source has a central role in corneal endothelial function by buffering corneal endothelial fluid transport. Carbonic anhydrases support one-third of active ion transport in bovine corneal endothelium but do not in human^16,17^.

Thiosemicarbazones are synthesized by the use of simple and economical methods through the condensation reaction of thiosemicarbazides with various aldehydes and ketones^18^. They are a type of N, S-donor ligands, possessing an important position in therapeutic chemistry research because of their structural assortment, modifiable synthesis, and valuable donating ability^19^. Thiosemicarbazones and their derivatives have a significant awareness in the organic field, pharmacology, and biology; appreciations to their various applications^20–29^. Several pieces of research have testified the antiviral, antioxidant^30^, antitumor, antimalarial^31,32^, antibacterial^33,34^, antifungal^35,36^, anticancer^37–41^, antiviral^42,43^ and anti-diabetic^44–46^ actions of thiosemicarbazones^47^. Moreover, the reputation of thiosemicarbazones has been much improved by the entry of α-*N*-heterocyclic TSCs derivates (Tripane, COTI-2, DpC) in the medical hearings for cancer and HIV-1^48^.

Biological interaction has been upholding one of the greatest substantial goals in the growth of human strength i.e., providing organically energetic unrelated molecules that can be found over simple synthetic ways with the non-poisonous catalysts and can be reformed and improved for better pharmacological offers, in the midst are thiosemicarbazones (TSCs). The pharmacological properties of thiosemicarbazones assets are due to the presence of thiourea based functional nucleus, having NH and C=S functional groups for metallic coordination^49^. Research has revealed that therapeutic characteristics of thiosemicarbazones can be enhanced by utilizing vigorous biological moieties as the building blocks of molecules^50^.

The main chemical constituent obtained from *Cinnamomum cassia* is cinnamaldehyde^51^. In the earlier research, cinnamaldehyde has displayed noticeable characteristics in persuading in vivo or in vitro apoptosis of numerous tumor cells^52^. Our preceding research has exposed that cinnamaldehyde can persuade non-small cell lung cancer (NSCLC) apoptosis and control of Wnt/b-catenin path in A549, YTMLC-90, and H1299 cells beneath the conditions of cinnamaldehyde. Despite all these signs, additional studies are required to offer awareness into the possible anti-tumor mechanism complications in the cinnamaldehyde regulation. It is expected that the cinnamaldehyde derivatives might display extra energy anti-FtsZ result with a difficult antibacterial worth and a larger antibacterial spectrum^53,54^.

**Figure 1:**
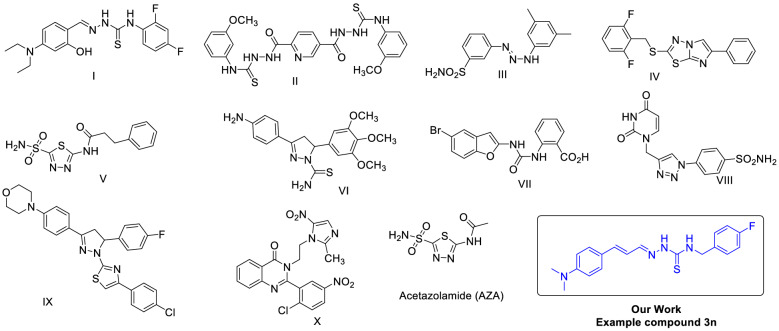
Examples of some known CA-II inhibitors with rational design of current work^29,59–67^.

Recent studies have also shown out the pathogenetic characters of oxidative strain in vitiligo and bad skin, both of which are conditions complex to ecological roughness**.** Furthermore, cinnamaldehyde exhibited strong antioxidant activity consequently since its activation of the NRF2/HO1 pathway. These representatives are gifted candidates for the action of oxidative stress-related sicknesses^55,56^. There have been some bits of intelligence on cinnamon oil impeding the development of shapes**,** cinnamaldehyde hydroxyl sulfonic sodium, which was non-volatile, stood also formed but displayed much lower anti-mold activity as compared to cinnamaldehyde. An assessable structure–activity association (QSAR) demonstrating the anti-mold activity of cinnamaldehyde correspondents against *Aspergillus niger* and *Paecilomyces variotii* stood offered^57,58^. There are numbers of sulfonamides derivatives are reported as CA-II inhibitors^29,59–67^. Some examples of known CA-II inhibitors are given in Fig. 1.

Recently, the inhibitory potential of thiosemicarbazones also reported for bovine and human CA-II^68^. We hereby report the syntheses of a novel 4-(dimethylamino)-cinnamaldehyde-based thiosemi-carbazones derivatives as potent CA-II inhibitors. Moreover, molecular docking studies were also carried to know the interaction of these derivatives at atomic level. We concluded that most of the compounds exhibited a stronger inhibitory effect as compared to acetazolamide which was used as a reference.

## Experimental

### Chemistry

All the chemicals used in this research were bought in extra purified from Sigma-Aldrich. FT-IR spectra (KBr disks) were recorded on a Bruker FT-IR IFS48 spectrophotometer. Melting points were taken on the melting point apparatus (Büchi 434). The CHN analysis was done on a Carlo Erba Strumentazione-Mod-1106. NMR spectra were taken on Bruker Avance 400 spectrometers in DMSO-d^6^. The chromatographs were seen under UV light irradiation.

### General method for the synthesis of thiosemicarbazones (3a–q)

A series of seventeen TSCs **(3a–q)** was prepared by adding suitable thiosemicarbazide **(2a–q)** (5 mmol) and 4-(dimethylamino)-cinnamaldehyde (**1)** (5 mmol) in 10 mL of methanol and glacial acetic acid in catalytic quantity. After refluxing for 3–6 h at 80 °C, the reaction progress was observed by the TLC analysis. The reaction mixture was left to cool at room temperature and the solid products obtained in all cases were filtered, washed with hot methanol, and dried at room temperature. The targeted TSCs were further recrystallized from the methanol-chloroform mixture (1:1) in very good to excellent yields.

The targeted compounds are characterized as follows:

#### *(E*)-2-((*E*)-3-(4-(Dimethylamino)phenyl)allylidene)-*N*-(*p*-tolyl)hydrazine-1-carbothioamide (3a)

Yield: 97%, color: brownish yellow; M.P: 232 °C; ^1^H NMR (DMSO-*d*^*6*^, δ, ppm): 2.28 (s, 3H, Ar–CH_3_), 2.93 (s, 6H, CH_3_), 6.63–6.72 (m, 3H, C=CH, Ar–H), 6.9 (d, *J* = 16.2 Hz, 1H, Ar–CH), 7.11 (d, *J* = 7.8 Hz, 2H, Ar–H), 7.38 (d, *J* = 9.0 Hz, 2H, Ar–H), 7.45 (d, *J* = 8.4 Hz, 2H, Ar–H), 7.91 (d, *J* = 9.6 Hz, 1H, N=CH), 9.7 (s, 1H, NH–CS), 11.59 (s, 1H, N–NH–CS). ^13^C NMR (δ, ppm): 20.6, 39.5, 112.1, 119.9, 123.6, 124.7, 128.4, 128.6, 134.0, 136.5, 140.3, 146.1, 150.8, 174.9, FT-IR (υ_max_, cm^−1^): 1184 (C=S), 1580 (C=N), 3290 (N–H); ESI MS (m/z) = M+H = 339.16.

#### (*E*)-2-((*E*)-3-(4-(Dimethylamino)phenyl)allylidene)-*N*-(2,6-dimethylphenyl)hydrazine-1-carbothioamide (3b)

Yield: 77%, color: yellow; M.P: 239 °C; ^1^H NMR (DMSO-*d*^*6*^, δ, ppm): 2.1 (s, 6H, Ar–CH_3_), 2.93 (s, 6H, CH_3_), 6.64–6.67 (m, 1H, C=CH, Ar–H), 6.7 (d, *J* = 8.4 Hz, 2H, Ar–H), 6.89 (d, *J* = 16.2 Hz, 1H, Ar–CH), 7.05–7.07 (m, 3H, Ar–H), 7.37 (d, *J* = 8.4 Hz, 2H, Ar–H), 7.91 (d, *J* = 9.6 Hz, 1H, N=CH), 9.44 (s, 1H, NH–CS), 11.51 (s, 1H, N–NH–CS). ^13^C NMR (δ, ppm): 18.1, 39.5, 112.1, 120.9, 123.7, 126.8, 127.6, 128.3, 136.4, 137.2, 139.8, 145.8, 150.7, 176.1, FT-IR (υ_max_, cm^−1^): 1186 (C=S), 1581 (C=N), 3292 (N–H); ESI MS (m/z) = M+H = 353.17.

#### (*E*)-2-((*E*)-3-(4-(Dimethylamino)phenyl)allylidene)-*N*-(4-ethylphenyl)hydrazine-1-carbothioamide (3c)

Yield: 75%, color: orange yellow; M.P: 180 °C; ^1^H NMR (DMSO-*d*^*6*^, δ, ppm): 2.87 (t, 2H, Ar–CH_2_), 2.93 (s, 6H, CH_3_), 3.71–3.74 (m, 2H, N–CH_2_), 6.58–6.62 (m, 1H, C=CH), 6.68 (d, *J* = 8.4 Hz, 2H, Ar–H), 6.84 (d, *J* = 15.6 Hz, 1H, Ar–CH), 7.20 (t, *J* = 7.2 Hz, 1H, Ar–H), 7.24 (d, *J* = 9.6 Hz, 2H, Ar–H), 7.30 (t, *J* = 7.2 Hz, 2H, Ar–H), 7.36 (d, *J* = 8.4 Hz, 2H, Ar–H), 7.84 (d, *J* = 9.0 Hz, 1H, N=CH), 8.17 (s, 1H, NH–CS), 11.28 (s, 1H, N–NH–CS). ^13^C NMR (δ, ppm): 34.9, 39.5, 44.9, 112.1, 119.8, 123.6, 126.2, 128.3, 128.4, 128.6, 139.3, 139.8, 145.6, 150.7, 176.3, FT-IR (υ_max_, cm^−1^): 1183 (C=S), 1579 (C=N), 3289 (N–H); ESI MS (m/z) = M+H = 353.18.

#### (*E*)-*N*-(2,3-Dichlorophenyl)-2-((*E*)-3-(4-(dimethylamino)phenyl)allylidene)hydrazine-1-carbothioamide (3d)

Yield: 95%, Color: Brownish Yellow; M.P: 192 °C; ^1^H NMR (DMSO-*d*^*6*^, *δ,* ppm): 2.98 (s, 6H, CH_3_), 6.62–6.67 (m, 3H, C=CH, Ar–H), 6.84 (d, *J* = 15.6 Hz, 1H, Ar–CH), 7.21–7.24 (m, 1H, Ar–H), 7.27 (d, *J* = 7.8 Hz, 1H, Ar–H), 7.34 (d, *J* = 9.0 Hz, 2H, Ar–H), 7.69 (d, *J* = 9.6 Hz, 1H, N=CH), 8.49 (d, *J* = 7.8 Hz, 1H, Ar–H), 9.57 (s, 1H, NH–CS), 10.03 (s, 1H, N–NH–CS). ^13^C NMR (δ, ppm): 40.2, 77.1, 111.1, 119, 123.5, 125.6, 126.7, 128.8, 129.7, 132.8, 136.9, 142.4, 146.9, 151.2, 174.4, FT-IR (υ_max_, cm^−1^): 1180 (C=S), 1575 (C=N), 3284 (N–H); ESI MS (m/z) = M+H = 393.07.

#### (*E*)-2-((*E*)-3-(4-(Dimethylamino)phenyl)allylidene)-*N*-(2-fluorophenyl)hydrazine-1-carbothioamide (3e)

Yield: 75%, color: yellow; M.P: 189 °C; ^1^H NMR (DMSO-*d*^*6*^, δ, ppm): 2.98 (s, 6H, CH_3_), 6.62–6.67 (m, 3H, C=CH, Ar–H), 6.83 (d, *J* = 15.2 Hz, 1H, Ar–CH), 7.11–7.17 (m, 3H, Ar–H), 7.34 (d, *J* = 8.4 Hz, 2H, Ar–H), 7.64 (d, *J* = 8.4 Hz, 1H, N=CH), 8.41–8.44 (m, 1H, Ar–H), 9.21 (s, 1H, NH–CS), 9.66 (s, 1H, N–NH–CS). ^13^C NMR (δ, ppm): 40.2, 77.0, 111.9, 115.3, 119.1, 123.5, 125.4, 126.2, 126.3, 128.7, 142.0, 146.4, 151.1, 154.1, 174.9, FT-IR (υ_max_, cm^−1^): 1181 (C=S), 1577 (C=N), 3286 (N–H); ESI MS (m/z) = M+H = 343.13.

#### (*E*)-2-((*E*)-3-(4-(Dimethylamino)phenyl)allylidene)-*N*-(3-methoxyphenyl)hydrazine-1-carbothioamide (3f)

Yield: 85%, color: brownish yellow; M.P: 198 °C; ^1^H NMR (DMSO-d^6^, δ, ppm): 2.98 (s, 6H, CH_3_), 3.8 (s, 3H, O-CH_3_), 6.62–6.6 (m, 3H, C=CH, Ar–H), 6.73 (d, *J* = 7.8 Hz, 1H, Ar–H) 6.82 (d, *J* = 15.6 Hz, 1H, Ar–CH), 7.14 (d, *J* = 8.4 Hz, 1H, Ar–H), 7.23–7.26 (t, *J* = 7.8 Hz, 1H), 7.34 (d, *J* = 8.4 Hz, 2H, Ar–H), 7.44 (s, 1H, Ar–H), 7.67 (d, *J* = 9.6 Hz, 1H, N=CH), 9.12 (s, 1H, NH–CS), 9.95 (s, 1H, N–NH–CS). ^13^C NMR (δ, ppm): 40.2, 65.4, 77.1, 109.1, 110.1, 116.1, 119.1, 123.7, 128.7, 129.3, 139.2, 141.9, 146.2, 151.1, 159.8, 174.6, FT-IR (υ_max_, cm^−1^): 1187 (C=S), 1587 (C=N), 3297 (N–H); ESI MS (m/z) = M+H = 355.16.

#### (*E*)-*N*-(4-Bromophenyl)-2-((*E*)-3-(4-(dimethylamino)phenyl)allylidene)hydrazine-1-carbothioamide (3g)

Yield: 80%, color: brownish yellow; M.P: 240 °C; ^1^H NMR (DMSO-d^6^, δ, ppm): 2.93 (s, 6H, CH_3_), 6.60–6.64 (m, 3H, C=CH, Ar–H), 6.78 (d, *J* = 16.2 Hz, 1H, Ar–CH) 7.27 (d, *J* = 8.4 Hz, 2H, Ar–H), 7.36 (d, *J* = 7.8 Hz, 2H, Ar–H), 7.58 (d, *J* = 8.4 Hz, 2H, Ar–H), 7.86 (d, *J* = 9.6 Hz, 1H, N=CH), 9.49 (s, 1H, NH–CS), 11.59 (s, 1H, N–NH–CS). ^13^C NMR (δ, ppm): 39.7, 78.3, 111.7, 117.1, 119.6, 123.5, 128.1, 130.7, 137.9, 140.4, 146.4, 150.6, 174.4, FT-IR (υ_max_, cm^−1^): 1185 (C=S), 1582 (C=N), 3291 (N–H); ESI MS (m/z) = M+H = 403.6.

#### (*E*)-*N*-(2,4-Difluorophenyl)-2-((*E*)-3-(4-(dimethylamino)phenyl)allylidene)hydrazine-1-carbothioamide (3h)

Yield: 95%, color: brownish yellow; M.P: 202 °C; ^1^H NMR (DMSO-d^6^, δ, ppm): 2.98 (s, 6H, CH_3_), 6.62–6.65 (m, 3H, C=CH, Ar–H), 6.83–6.90 (m, 3H, Ar–CH, Ar–H), 7.33 (d, *J* = 8.4 Hz, 2H, Ar–H), 7.67 (d, *J* = 9.0 Hz, 1H, N=CH), 8.17–8.21 (m, 1H, Ar–H), 9.0 (s, 1H, NH–CS), 9.94 (s, 1H, N–NH–CS). ^13^C NMR (δ, ppm): 40.2, 77.0, 103.9, 110.0, 112.0, 119.0, 122.7, 123.7, 127.5, 128.7, 129.7,142.2, 146.7, 151.2, 175.4, FT-IR (υ_max_, cm^−1^): 1182 (C=S), 1576 (C=N), 3286 (N–H); ESI MS (m/z) = M+H = 361.12.

#### (*E*)-2-((*E*)-3-(4-(Dimethylamino)phenyl)allylidene)-*N*-(4-isopropylphenyl)hydrazine-1-carbothioamide (3i)

Yield: 70%, color: dark brown; M.P: 220 °C; ^1^H NMR (DMSO-d^6^, δ, ppm): 1.22 (d, *J* = 7.2 Hz, 6H, CH_3_), 2.8 (m, 1H, CH_3_-CH), 2.98 (s, 6H, CH_3_), 6.64 (m, 3H, C=CH, Ar–H), 6.82 (d, *J* = 15.6 Hz, 1H, Ar–CH), 7.21 (d, *J* = 7.8 Hz, 2H, Ar–H), 7.33 (d, *J* = 8.4 Hz, 2H, Ar–H), 7.54 (d, *J* = 8.4 Hz, 2H, Ar–H), 7.66 (d, *J* = 9.6 Hz, 1H, N=CH,), 9.04 (s, 1H, NH–CS), 9.81 (s, 1H, N–NH–CS). ^13^C NMR (δ, ppm): 24.02, 33.7, 40.2, 77.0, 112.0, 119.1, 123.8, 124.3, 126.7, 128.6, 135.7, 141.7, 146.5, 151.1, 174.9, FT-IR (υ_max_ (cm^−1^): 1195 (C=S), 1592 (C=N), 3298 (N–H); ESI MS (m/z) = M+H = 367.1.

#### (*E*)-2-((*E*)-3-(4-(Dimethylamino)phenyl)allylidene)-*N*-(3-fluorophenyl)hydrazine-1-carbothioamide (3j)

Yield: 85%, Color: Yellow; M.P: 218 °C; ^1^H NMR (DMSO-d^6^, δ, ppm): 2.99 (s, 6H, CH_3_), 6.63- 6.67 (m, 3H, C=CH, Ar–H), 6.84–6.89 (m, 2H, Ar–H, Ar–CH), 7.28 (dd, *J* = 7.2 Hz, 7.8 Hz, 1H, Ar–H), 7.34–7.37 (m, 3H, Ar–H), 7.63 (d, *J* = 9.0 Hz, 1H Ar–H), 7.67 (d, *J* = 10.2 Hz, 1H, N=CH), 9.16 (s, 1H, NH–CS), 9.58 (s, 1H, N–NH–CS). ^13^C NMR (δ, ppm): 40.2, 77.2, 110.8, 119.2, 112.2, 118.8, 119.0, 123.6, 128.7, 129.7, 139.7, 142.1, 146.3, 151.2, 174.6, FT-IR υ_max_ (cm^−1^): 1182 (C=S), 1578 (C=N), 3289 (N–H); ESI MS (m/z) = M+H = 343.10.

#### (*E*)-*N*-(4-(Chloromethyl)phenyl)-2-((*E*)-3-(4-(dimethylamino)phenyl)allylidene)hydrazine-1-carbothioamide (3k)

Yield: 80%, color: orange yellow; M.P: 196 °C; ^1^H NMR (DMSO-d^6^, δ, ppm): 2.97 (s, 6H, CH_3_), 4.86 (d, *J* = 5.4 Hz, 2H, Ar–CH_2_), 6.53–6.57 (m, 1H, C=CH), 6.62 (d, *J* = 8.4 Hz, 2H, Ar–H), 6.77 (d, *J* = 15.6 Hz, 1H, Ar–CH), 7.29–7.30 (m, 6H, Ar–H), 7.59 (m, 2H, N=CH, NH–CS), 9.63 (s, 1H, N–NH–CS). ^13^C NMR (δ, ppm): 40.2, 47.4, 77.0, 112.0, 119.1, 123.7, 128.6, 128.8, 129.2, 136.3, 141.4, 146.2, 151.0, 177.0, FT-IR (υ_max_, cm^−1^): 1183 (C=S), 1580 (C=N), 3290 (N–H); ESI MS (m/z) = M+H = 373.12.

#### (*E*)-2-((*E*)-3-(4-(Dimethylamino)phenyl)allylidene)-*N*-(2,4-dimethylphenyl)hydrazine-1-carbothioamide (3l)

Yield: 85%, color: yellow; M.P: 232 °C; ^1^H NMR (DMSO-d^6^, δ, ppm): 2.27 (s, 3H, Ar-CH_3)_, 2.30 (s, 3H, Ar-CH_3_), 2.98 (s, 6H, CH_3_), 6.63- 6.65 (m, 3H, C=CH, Ar–H), 6.81 (d, *J* = 16.2 Hz, 1H, Ar–CH), 7.02–7.05 (m, 2H, Ar–H), 7.32 (d, *J* = 9.0 Hz, 2H, Ar–H), 7.40 (d, *J* = 7.8 Hz, 1H Ar–H), 7.66 (d, *J* = 9 Hz, 1H, N=CH), 8.74 (s, 1H, NH–CS), 9.81 (s, 1H, N–NH–CS). ^13^C NMR (δ, ppm): 17.9, 21.1, 40.2, 77.2, 112.0, 119.2, 123.8, 127.4, 128.6, 129.5, 131.3, 134.2, 137.0, 141.5, 146.0, 151.1, 176.3, FT-IR (υ_max_, cm^−1^): 1197 (C=S), 1595 (C=N), 3297 (N–H); ESI MS (m/z) = M+H = 353.17.

#### (*E*)-*N*-(4-Chlorophenyl)-2-((*E*)-3-(4-(dimethylamino)phenyl)allylidene)hydrazine-1-carbothioamide (3m)

Yield: 90%, color: brownish yellow; M.P: 238 °C; ^1^H NMR (DMSO-d^6^, δ, ppm): 2.90 (s, 6H, CH_3_), 6.54–6.58 (m, 3H, C=CH, Ar–H), 6.60 (d, *J* = 15.6 Hz, 1H), 7.19 (d, *J* = 8.4 Hz, 2H, Ar–H), 7.23 (d, *J* = 8.4 Hz, 2H, Ar–H), 7.56 (d, *J* = 8.4 Hz, 2H, Ar–H), 7.80 (d, *J* = 9.0 Hz, 1H, N=CH), 9.2 (s, 1H, NH–CS), 11.3 (s, 1H, N–NH–CS). ^13^C NMR (δ, ppm): 40.0, 77.7, 112.0, 119.8, 123.8, 125.4, 128.5, 130.0, 137.4, 141.1, 146.9, 150.9, 174.9, FT-IR (υ_max_, cm^−1^): 1185 (C=S), 1579 (C=N), 3289 (N–H); ESI MS (m/z) = M+H = 359.11.

#### (*E*)-2-((*E*)-3-(4-(Dimethylamino)phenyl)allylidene)-*N*-(4-(fluorobenzyl)hydrazine-1-carbothioamide (3n)

Yield: 80%, color: brownish yellow; M.P: 192 °C; ^1^H NMR (DMSO-d^6^, δ, ppm): 2.96 (s, 6H, CH_3_), 4.86 (d, *J* = 4.8 Hz, 2H, Ar–CH_2_), 6.55–6.58 (m, 1H, C=CH, Ar–H), 6.62 (d, *J* = 7.8 Hz, 2H, Ar–H), 6.78 (d, *J* = 15.6 Hz, 1H, Ar–CH), 6.99–7.02 (m, 2H, Ar–H), 7.28–7.33 (m, 4H, Ar–H), 7.59–7.62 (m, 2H, NH–CS, N=CH), 9.7 (s, 1H, N–NH–CS). ^13^C NMR (δ, ppm): 40.2, 47.5, 111.9, 115.5 (*I*_CF_ = 85.32 Hz), 119.2, 123.7, 128.6, 129.5(*I*_CF_ = 26.95 Hz), 133.5(*I*_CF_ = 10.05 Hz), 146.1, 151.0, 161.5, 163.1, 176.9, FT-IR (υ_max_, cm^−1^): 1183 (C=S), 1589 (C=N), 3286 (N–H); ESI MS (m/z) = M+H = 357.15.

#### (*E*)-*N*-(3-Cyanophenyl)-2-((*E*)-3-(4-(dimethylamino)phenyl)allylidene)hydrazine-1-carbothioamide (3o)

Yield: 70%, color: brownish yellow; M.P: 215 °C; ^1^H NMR (DMSO-d^6^, δ, ppm): 2.98 (s, 6H, CH_3_), 6.60–6.64 (m, 3H, C=CH, Ar–H), 6.86 (d, *J* = 15.6 Hz, 1H, Ar–CH), 7.33 (d, *J* = 8.4 Hz, 2H, Ar–H), 7.43 (d, *J* = 5.4 Hz, 2H, Ar–H,), 7.71 (d, *J* = 9.0 Hz, 1H, N=CH), 7.92–7.93 (m, 1H, Ar–), 8.09 (s, 1H, Ar–H), 9.2 (s, 1H, NH–CS), 10.21 (s, 1H, N–NH–CS). ^13^C NMR (δ, ppm): 40.2, 77.1, 112.0, 112.6, 118.5, 118.6,123.5, 126.8, 128.0,128.8, 129.5, 139.1, 142.7, 147.1, 151.2, 174.4, FT-IR (υ_max_, cm^−1^): 1187 (C=S), 1583 (C=N), 3281 (N–H); ESI MS (m/z) = M+H = 350.14.

#### (*E)*-2-((*E*)-3-(4-(Dimethylamino)phenyl)allylidene)-*N*-phenylhydrazine-1-carbothioamide (3p)

Yield: 75%, color: orange yellow; M.P: 218 °C; ^1^H NMR (DMSO-d^6^, δ, ppm): 2.98 (s, 6H, CH_3_), 6.65–6.69 (m, 3H, C=CH, Ar–H), 6.84 (d, *J* = 15.6 Hz, 1H, Ar–CH), 7.10 (t, *J* = 7.2 Hz, 1H, Ar–H), 7.35–7.37 (m, 4H, Ar–H), 7.64–7.67 (m, 3H, Ar–H, N=CH), 9.1 (s, 1H, NH–CS), 9.7 (s, 1H, N–NH–CS). ^13^C NMR (δ, ppm): 40.2, 77.3, 112.1, 119.1, 124.0, 125.8, 128.7, 129.6, 138.1, 141.8, 146.1, 151.1, 174.9, FT-IR (υ_max_, cm^−1^): 1184 (C=S), 1588 (C=N), 3287 (N–H); ESI MS (m/z) = M+H = 325.14.

#### (*E*)-*N*-Cyclohexyl-2-((*E*)-3-(4-(dimethylamino)phenyl)allylidene)hydrazine-1-carbothioamide (3q)

Yield: 80%, color: pale yellow; M.P: 252 °C; ^1^H NMR (DMSO-d^6^, δ, ppm): 1.17–1.28 (m, 5H, Cyclohexyl), 1.37–1.43 (m, 2H, Cyclohexyl), 1.61–1.63 (m, 1H, Cyclohexyl), 1.71–1.75 (m, 2H, Cyclohexyl), 2.97 (s, 6H, CH_3_), 4.20–4.27 (m, 1H, Cyclohexyl), 6.58–6.65 (m, 3H, C=CH, Ar–H), 6.76 (d, *J* = 17.4 Hz, 1H, Ar–CH), 7.20 (t, *J* = 8.4 Hz, 1H, NH–CS), 7.54 (d, *J* = 9.0 Hz, 2H, N=CH), 9.25 (s, 1H, N–NH–CS). ^13^C NMR (δ, ppm): 25.5, 32.8, 40.2, 53.0, 77.0, 111.9, 119.4, 123.9, 128.5, 140.9, 145.4, 151.0, 175.3, FT-IR (υ_max_, cm^−1^): 1181 (C=S), 1586 (C=N), 3288 (N–H); ESI MS (m/z) = M+H = 331.19.

### Carbonic anhydrase II (CA-II) inhibition protocol

Evaluation of carbonic anhydrase inhibition was done by spectrophotometric technique as narrated by Pocker and Meany with little alteration^69,70^ as described previously^3^. The process was carried out in HEPES-Tris buffer (20 mM) having pH 7.4 at 25 °C. Each inhibitory well contained 20 µL of CA-II enzyme solution (0.1 mg/mL HEPES-Tris buffer), 140 µL of HEPES-Tris buffer solution, 20 µL of the compound to be analyzed in HPLC grade DMSO. At 25 °C, the solution mixture was pre-incubated for 15 min. Substrate p-nitrophenyl acetate (p-NPA) (0.7 mM) was prepared in HPLC grade methanol and the reaction was initiated by the addition of 20 µL to a well in a 96-well-plate. The quantity of product formed was measured at 400 nm continually at 1 min gap for 30 min in a 96-well-plate using xMARK microplate spectrophotometer, Bio-Rad (USA). The inhibition activity of the controlled compound was considered as 100%. All the experiments were performed in triplicates of each used concentration, and the conclusions are expressed as a mean of the triplicate.

### Molecular docking

The X-ray crystal structure of human carbonic anhydrase II in complex with the standard drug, acetazolamide (PDB code: 3HS4, resolution: 1.10 Å) was selected from RCSB-Protein databank for molecular docking^71^, which was performed on Molecular Operating Environment (MOE version 2020.0901)^72^. Previously, we have carried out re-docking to test the docking performance of MOE and MOE showed outstanding result^73–75^. In this work, the protein file was prepared for docking by adding missing hydrogen atoms on protein by QuickPrep module of MOE. QuickPrep also calculates partial charges using Amber10:EHT force field. The two-dimensional structures of active CA-II inhibitors were drawn by ChemDraw and imported into MOE database. In MOE database, MOE-WASH module was used to convert 2D strictures into three-dimensional format by which all hydrogen atoms and partial charges on all the compounds and minimize the structure of each ligand with RMS gradient of 0.1RMS kcal/mol/Å. The docking was performed with Triangle Matcher docking algorithm and London dG scoring function. After docking, the best docked conformation of each ligand was selected based on good docking score and good binding interaction.

## Results and discussion

### Chemistry

To discover the biological potential of 4-(dimethylamino)-cinnamaldehyde-based thiosemicarbazones (**3a–q**), a thiosemicarbazones (**TSC**) series bearing cycloalkyl and aryl substituents were synthesized. A classic method of condensation was adopted in which various thiosemicarbazides (**2a–q**) were reacted with 4-(dimethylamino)-cinnamaldehyde (**1**). The reaction was conducted in methanol using 1–2 drops of glacial acetic acid. The optimum conditions were set by the reaction of phenyl thiosemicarbazide (**16**) and 4-dimethylamino cinnamaldehyde (**1**) in a 1:1 ratio and by shifting the polarity of solvent from polar to non-polar i.e., methanol, DMSO, ethanol, THF, and DCM. The reaction conditions were optimized by using methanol as solvent and catalytic amount of glacial acetic acid (1-2 drops). The extent of the reaction was investigated and expanded by utilizing thiosemicarbazides (**2a–q**) with various cycloalkyl, heterocyclic, and aryl substituents at the N-4 position of thiosemicarbazides. The aimed compounds (**3a–q**) were obtained in good to outstanding yields (46–98%).

The structures of 4-(dimethylamino)-cinnamaldehyde conjugates of N-4 thiosemicarbazides (**3a–q**) were established by using spectral data i.e., IR, ^1^H-NMR, ^13^C-NMR (S.I. 1–34), microanalysis (CHN), and ESI spectrometry. In FTIR, the signal for C=S appeared in the 1177–1246 cm^−1^ range while the C=N band for several derivatives appeared ranging from 1484 to 1605 cm^−1^. N–H stretching appeared in the 3162–3469 cm^−1^ range. In the ^1^HNMR, the N=CH signal was seen as a doublet at *δ* 7.54–7.91 ppm, though the N–NH–C=S appeared as a singlet at *δ* 9.21–11.59 ppm; and CH_3_ linked with N-CH_2_ was seen as a triplet in the range of *δ* 1.08–1.11 ppm. The NH–CS proton displayed variable behavior and appeared as a singlet in the range of *δ* 9.04–9.49 ppm when directly attached to an aromatic ring. While, in the compounds **3c, 3k,** and **3n,** the NH–CS appeared as a multiplet in the range of *δ:* 7.59–7.63 ppm because of the presence of neighboring CH_2_-group while in TSC **3q** bearing *N*-4 cyclohexyl substituent, a doublet appeared for NH-CS at *δ:* 7.20 ppm (*J* = 8.4 Hz). The spectral details of other aromatic and aliphatic protons were also following the proposed compounds. In ^13^C NMR spectra of 3n, owing to the presences of F, coupling of C-F was also observed.

### In-vitro inhibition of *b*CA-II and structure activity relationship (SAR)

All thiosemicarbazones derivatives **(3a–q)** were tested against bovine carbonic anhydrase II (*b*CA-II). The assay was conducted at µM level and acetazolamide was used as a reference inhibitor. The novel derivatives displayed effective inhibition of *b*CA-II, with inhibition constant (IC_50_) ranging from 10.3 ± 0.62–46.6 ± 0.62 µM except for **3j**, **3p**, and **3q** which showed activity less than 50% (Table 1). Additionally, the compounds of interest **3f**, **3g**, **3h**, **3m**, and **3n** displayed higher inhibitory activity than the standard drug, acetazolamide (IC_50_ = 18.2 ± 0.43 µM). Among all the synthesized compounds, the best *b*CA-II inhibitor was **3n** (IC_50_ = 10.3 ± 0.62 µM), followed by **3g** (IC_50_ = 12.1 ± 1.01 µM), **3h** (IC_50_ = 13.4 ± 0.52 µM), **3m** (IC_50_ = 14.7 ± 1.42 µM) and **3f** (IC_50_ = 17.9 ± 1.23 µM). While compounds **3l**, **3e** and **3o** depicted inhibitory activities like acetazolamide with IC_50_ values in range of 18.3 ± 1.32–21.8 ± 0.85 µM. Moreover, **3i**, **3d**, **3c**, **3b**, **3a** and **3k** were found as least active inhibitors with IC_50_ in range of 33.6–49.6 µM**.**

**Table 1:** In vitro *b*CA-II inhibition values of synthesized compounds (**3a–q**)*.*


Bovine carbonic anhydrase-II (*b*CA-II)			
Compounds	R	% Inhibtion (500 µM)	IC_50_ ± SEM (µM)
**3a**	*p*-Tolyl	82.0	46.6 ± 0.62
**3b**	2,6-Dimethylphenyl	78.1	40.4 ± 0.89
**3c**	4-Ethylphenyl	75.9	39.7 ± 0.56
**3d**	2,3-Dichlorophenyl	82.2	36.6 ± 0.15
**3e**	2-Fluorophenyl	93.4	19.7 ± 0.23
**3f**	3-Methoxyphenyl	89.7	17.9 ± 1.23
**3g**	4-Bromophenyl	93.0	12.1 ± 1.01
**3h**	2,4-Difluorophenyl	77.1	13.4 ± 0.52
**3i**	4-Isopropylphenyl	84.9	33.6 ± 0.42
**3j**	3-Fluorophenyl	38.4	NA
**3k**	4-Chlorobenzyl	80.3	49.6 ± 1.06
**3l**	2,4-Dimethylphenyl	78.2	18.3 ± 1.32
**3m**	4-Chlorophenyl	79.9	14.7 ± 1.42
**3n**	4-Fluorobenzyl	63.2	10.3 ± 0.62
**3o**	3-Cyanophenyl	81.8	21.8 ± 0.85
**3p**	Phenyl	28.0	NA
**3q**	Cyclohexyl	10.6	NA
Acetazolamide		86.4	18.2 ± 0.43

**Scheme 1:**
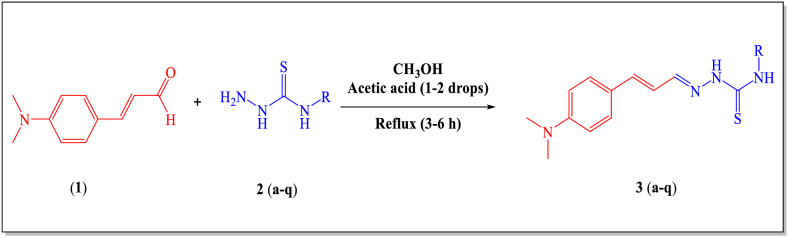
The synthetic route of compounds **3(a–q).**

The preliminary SAR suggested that the compounds showed inhibitory activity mainly due to the presence of thiosemicarbazide moiety. However, we further investigate the effect of various substitutions on the phenyl ring of the thiosemicarbazide. It can be seen in Scheme 1, the derivatives **3n** (4-fluorobenzyl), **3g** (4-bromophenyl) and **3h** (4-fluorophenyl), showed superior activity, as compared to acetazolamide, and other derivatives of this series. It indicates that electron donating groups (i.e., flouro and bromo) at the 4-position of phenyl or benzyl rings are highly important for the potent activity of these compounds against *b*CA-II. It was further proved when the activity of **3n** was compared with the biological activity of **3a** (4-CH_3_). A significant decrease in the inhibitory potential of **3a** indicates the importance of electronegative atom at para position of substituted phenyl ring. Whereas other derivatives including **3l** (2, 4-CH_3_), **3e** (2-F) and **3o** (3-CN) substituted phenyl ring, respectively, showed comparable activity when compared to acetazolamide, however these compounds exhibited less potent inhibitory activity as compared to **3n**, **3g** and **3h**. As the variation occurred only at “R” position, thus we could generate limited SAR. The substituents on phenyl ring reflects the importance of position with regards to the inhibitory activities of compounds. As we noted in derivatives **3e** (2-F) and **3j** (3-F) the position of fluorine atom is changed, thus significant variation in the activity was observed.

Whereas compounds **3a** (4-CH_3_), **3b** (2, 6-CH_3_), **3c** (4-C_2_H_5_), **3d** (2, 3-Cl), **3i** (4-C_3_H_7_) and **3k** (4-CH_2_Cl) on the phenyl ring showed inferior activity as compared to acetazolamide. The SAR suggests that the nature of atom (either electron donating or electron withdrawing), and their position on the phenyl ring is highly important for the potent inhibitory activity of the compounds.

### Molecular docking studies

Computational docking was employed to study the binding behavior of compounds at the active site of CA-II. All the compounds were fitted at the entrance of the active site, thus block the access of the substrate, therefore inhibit the normal function of CA-II. The docked orientation of the most active compound **3n**, reflect that the dimethylamino-cinnamaldehyde moiety of the compound interact at the entrance of the enzyme, while the thiosemicarbazide group interacted with the side chain of Thr200 through H-bond, and the thio group mediated H-bond with the amino group of Thr199 and ionic interaction with Zn ion at the core of the active. The docking score (DS) of **3n** is highly negative (− 6.75 kcal/mol) suggesting the higher inhibitory potential of this compound against CA-II, which is correlated with our experimental result.

Subsequently, four compounds (**3g**, **3h**, **3m** and **3f**) exhibited higher inhibitory potency as compared to the standard compound, acetazolamide. The biological activity of these compounds was in range of 12.1–17.9 µM. The binding mode of **3g** depict that it adapted the same binding mode of **3n**, however its para substituted Br atom did not interacted with the surrounding residues due to steric hindrance, however, its thiosemicarbazide group mediated H-bonding with the side chains of Asn62 and Gln92, thus this compound exhibited inhibitory activity comparable with the biological activity of **3n**. The substitution of fluorine group at ortho-para position of compound **3h** caused the compound to tilt from core of active site towards hydrophobic residues (Trp5, His64 and Ala65). However, the thio group of **3h** was oriented towards the core where the side chain of Thr200 provided H-bond to the thio group, and its linked amino group formed a H-bond with the side chain of Gln92. These strong interactions are responsible for good inhibitory activity of this compound. Similar conformation was adapted by compound **3m**, which indicates that the addition of bulky group at substituted phenyl ring creates steric hindrance at the core of active site, as a result, phenyl ring bend towards the loop region (Trp5, His64 and Ala65). The carbazide amino group of **3m** retained H-bonding with the side chain of Gln92, however its thio group lost interaction with the Thr200. The binding orientation of compound **3f** was similar with the docked orientation of **3g**. The thio and the amino groups of **3f** were stabilized by the side chains of Asn62 and Gln92, respectively by H-bonding, however its substituted methoxy group at meta position of phenyl ring did not interact with the active residues. The compound **3l** exhibited the inhibitory activity comparable to the acetazolamide. The binding mode of **3l** was found similar with the docked conformations of **3g** and **3f**. The addition of dimethyl group at meta para positions of phenyl ring caused the compound **3l** to move more towards the entrance of the active site, however its thio group mediate bidentate interaction with the side chains of Asn62 and Asn67. The addition of fluorine at ortho position of phenyl ring of compound **3e** produced similar binding effects like **3h**. The fluorophenyl and cyanophenyl rings of **3e** and **3o**, respectively were tilted towards His64, and Ala65, however, the thio group of **3e** accepted two H-bonds with the side chains of Thr199 and Thr200, and the thiosemicarbazone moiety of **3o** formed strong H-bonding with the side chains of Gln92 and Thr200. In addition, the side chain of His94 provided hydrophobic interaction to the substituted phenyl rings of both the compounds.

**Figure 2:**
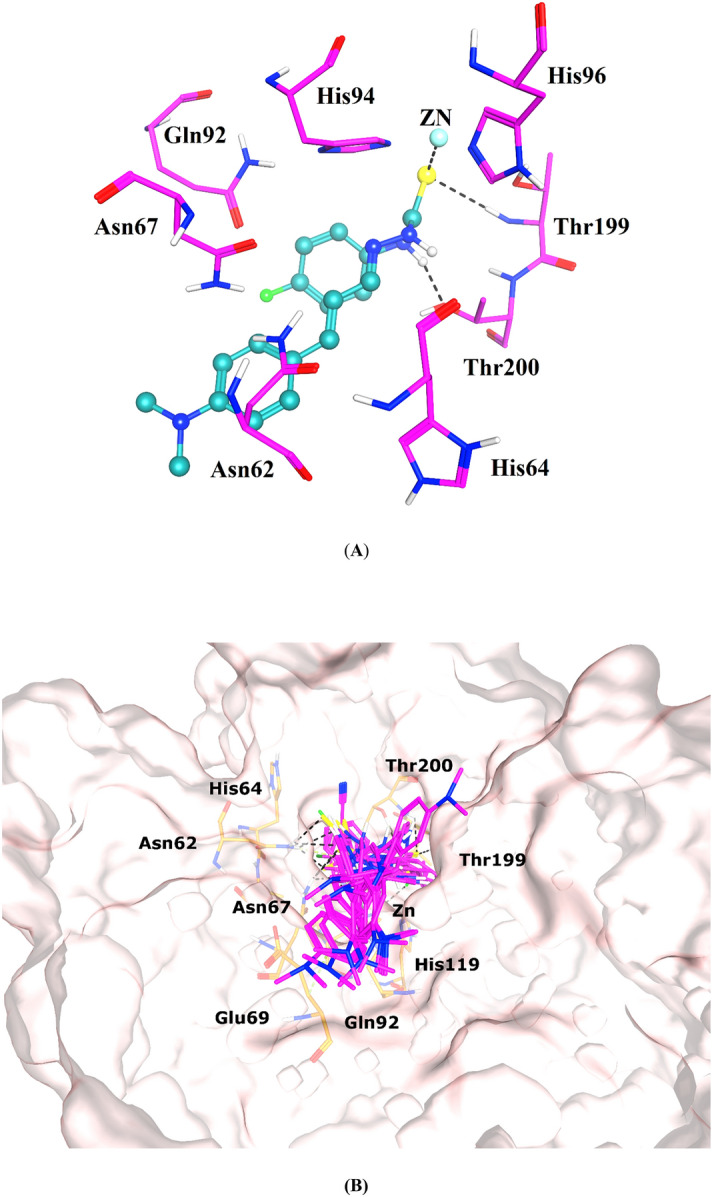
(**A**) The binding mode of the most active compound (**3n**) is shown. The ligand is depicted in cyan ball and stick model, interacting residues are demonstrated in magenta stick model, and hydrogen bonds are presented in black dotted lines. (**B**) The docked conformations of all the active ligands are displayed in the active site of CA-II. Ligands are presented in magenta color, the active site residues are shown in orange stick, H-bonds are depicted in black dotted lines. The protein in shown in surface model.

The compounds **3i**, **3d**, and **3c** exhibited inhibitory potential with IC_50_ values in range of 33.6–39.7 µM, while compounds **3b**, **3a** and **3k** displayed lowest inhibitory activities with IC_50_ ranging from 40.4 to 49.6 µM. The observed binding modes of these compounds (**3i**, **3d**, **3c**, **3b**, **3a** and **3k**) reflect that those compounds mediated only one H-bond interaction either with the side chain of Gln92, Asn62 or Asn67, thus the loss of higher number of H-bonds with surrounding residues or ionic interaction with Zn ion in the active site could be the possible reason of lower activity of those compounds. Moreover, the binding score of these compounds are also in range of − 5.73 to − 4.42 kcal/mol, which is lowest score among all the compounds. The docking scores and the binding interactions of each docked ligand with the active site residues of *b*CA-II are given in Table 2. The docking results match with our in vitro results. The docked conformations of all the active inhibitors are displayed in Fig. 2.

**Table 2:** Molecular docking results of active compounds against *b*CA-II.

Comp	Score (kcal/mol)	Protein–ligand interactions			
		Ligand atom	Receptor atom	Interaction	Distance (Å)
**3n**	− 6.75	N31	OG1-THR200	HBD	2.26
		S30	N-THR199	HBA	3.08
		S30	ZN	Ionic	2.75
**3g**	− 6.15	N31	OE1-GLN92	HBD	2.41
		S30	ND2-ASN62	HBA	2.90
		6-ring	5-Ring-HIS94	Π–π	3.00
**3h**	− 6.12	N31	OE1-GLN92	HBD	2.34
		S30	OG1-THR200	HBA	3.28
		6-ring	CG2-THR200	π–H	3.23
**3m**	− 6.06	N31	OE1-GLN92	HBD	2.27
		6-ring	ND2-ASN62	π–H	2.81
**3f**	− 6.02	N18	OE1-GLN92	HBD	2.43
		S30	ND2-ASN62	HBA	2.42
**3l**	− 6.00	S30	ND2-ASN62	HBA	2.70
		S30	ND2-ASN67	HBA	2.53
		6-ring	CB-GLU69	π–H	3.23
**3e**	− 5.95	S30	N-THR199	HBA	2.56
		S30	N-THR200	HBA	2.92
		6-ring	5-Ring-HIS94	π–π	3.04
**3o**	− 5.83	N31	OE1-GLN92	HBD	2.46
		S30	OG1-THR200	HBA	2.85
		C41	5-Ring-HIS94	π–H	3.12
**3i**	− 5.73	N31	OE1-GLN92	HBD	2.33
**3d**	− 5.67	S30	ND2-ASN67	HBA	3.32
**3c**	− 5.59	N31	OE1-GLN92	HBD	2.10
**3b**	− 5.55	N31	OE1-GLN92	HBD	2.25
		6-ring	OG1-THR200	π–H	2.92
		6-ring	5-Ring-HIS94	π–π	2.86
**3a**	− 4.95	S30	ND2-ASN67	HBA	2.36
**3k**	− 4.42	S30	N-ASN62	HBA	2.51
**Azm**	− 6.08	N3	OG1-THR200	HBA	3.08
		O1	N-THR199	HBA	2.96
		N1	ZN	Ionic	2.05
		N1	ZN	Ionic	2.05
		O2	ZN	Ionic	3.25
		5-Ring	CD2-LEU198	π–H	3.68

*Azm* acetazolamide, *HBA* hydrogen bond acceptor, *HBD* hydrogen bond donor.

## Conclusion

In summary, we have designed, synthesized, and evaluated a series of thiosemicarbazide derivatives as carbonic anhydrase-II inhibitors. These compounds could be further investigated for the treatment of carbonic anhydrase related biological disorders. Several compounds showed potent activity (i.e., **3f**, **3g**, **3h**, **3l**, **3m**, and **3n**) in in vitro biochemical assay. The active molecules were scrutinized in silico by molecular docking method to study their mode of binding within the active site of *b*CA-II. We observed that the high active leads bind specifically with Gln92, Thr199, Thr200, Asn62, and Asn67 via H-bonding, thus significantly inhibit the enzyme activity. Based on the promising in vitro and in silico results, these lead molecules can be a good choice for the in vivo studies. The identified compounds can be used as a good template drug for the diseases associated with the hyperactivity of CA-II. Moreover, these compounds will be also checked on CA-II from human source to know their selectivity.

## Supplementary Information

Supplementary Information.

## Data Availability

All data generated or analyzed during this study are included in this published article [and its supplementary information files].
