# Supplementary material for: Bis-pharmacophore of cinnamaldehyde-clubbed thiosemicarbazones as potent carbonic anhydrase-II inhibitors
Source: Sci Rep. 2022 Sep 27;12:16095. doi: 10.1038/s41598-022-19975-y (PMC9515202; doi:10.1038/s41598-022-19975-y)
Supplement: Supplementary file 1 — Supplementary Information. [file 41598_2022_19975_MOESM1_ESM.docx]

**Supporting Information**

**Bis-pharmacophore of cinnamaldehyde-clubbed thiosemicarbazones as potent** **Carbonic anhydrase-II inhibitors**

Asif Rasool^a^, Zahra Batool^a^, Majid Khan^b^, Sobia Ahsan Halim^b^, Zahid Shafiq^a,c^*, Ahmed Temirak^d^, Mohamed A. Salem^e,f^, Tarik E. Ali^g,h^ Ajmal Khan^b^* and Ahmed Al-Harrasi^b^*

*^a^Institute of Chemical Sciences, Bahauddin Zakariya University, 60800 Multan, Pakistan*

*^b^Natural and Medical Sciences Research Center, University of Nizwa, Nizwa, Sultanate of Oman*

*^c^Department of Pharmaceutical & Medicinal Chemistry, An der Immenburg 4, D-53121 Bonn, Germany*

*^d^National Research Centre, Chemistry of Natural and Microbial Products Department, Pharmaceutical and Drug Industries Research Institute, Dokki, Cairo P.O. Box 12622, Egypt*

*^e^Department of Chemistry, Faculty of Science & Arts, King Khalid University, Mohail, Assir, KSA*

*^f^Department of Chemistry, Faculty of Science, Al-Azhar University, 11284 Nasr City, Cairo, Egypt;*

*^g^Department of Chemistry, Faculty of Science, King Khalid University, Abha, Saudi Arabia*

*^h^Department of Chemistry, Faculty of Education, Ain Shams University*

*Corresponding author:

Zahid Shafiq: e-mail: [zahidshafiq@bzu.edu.pk](mailto:zahidshafiq@bzu.edu.pk), Tel. +92-3006559811;

Ahmed Al-Harrasi: e-mail: [aharrasi@unizwa.edu.om](mailto:aharrasi@unizwa.edu.om), Tel. +96825446328;

Ajmal Khan: e-mail: [ajmalkhan@unizwa.edu.om](mailto:ajmalkhan@unizwa.edu.om), Tel. +96825446502

**^1^H & ^13^C NMR Spectra of Compounds (3a-3q)**

**S.I. 1** ^1^H NMR spectrum of **3a**

**S.I. 2** ^13^ CNMR spectrum of **3a**

**S.I. 3** ^1^H NMR spectrum of **3b**

**S.I. 4** ^13^ CNMR spectrum of **3b**

**S.I. 5** ^1^H NMR spectrum of **3c**

**S.I. 6** ^13^ CNMR spectrum of **3c**

**S.I. 7** ^1^H NMR spectrum of **3d**

**S.I. 8** ^13^ CNMR spectrum of **3d**

**S.I. 9** ^1^H NMR spectrum of **3e**

**S.I. 10** ^13^ CNMR spectrum of **3e**

**S.I. 11** ^1^H NMR spectrum of **3f**

**S.I. 12** ^13^ CNMR spectrum of **3f**

**S.I. 13** ^1^H NMR spectrum of **3g**

**S.I. 14** ^13^ CNMR spectrum of **3g**

**S.I. 15** ^1^H NMR spectrum of **3h**

**S.I. 16** ^13^ CNMR spectrum of **3h**

**S.I. 17** ^1^H NMR spectrum of **3i**

**S.I. 18** ^13^ CNMR spectrum of **3i**

**S.I. 19** ^1^H NMR spectrum of **3j**

**S.I. 20** ^13^ CNMR spectrum of **3j**

**S.I. 21** ^1^H NMR spectrum of **3k**

**S.I. 22** ^13^ CNMR spectrum of **3k**

**S.I. 23** ^1^H NMR spectrum of **3l**

**S.I. 24** ^13^ CNMR spectrum of **3l**

**S.I. 25** ^1^H NMR spectrum of **3m**

**S.I. 26** ^13^ CNMR spectrum of **3m**

**S.I. 27** ^1^H NMR spectrum of **3n**

**S.I. 28** ^13^ CNMR spectrum of **3n**

**S.I. 29** ^1^H NMR spectrum of **3o**

**S.I. 30** ^13^ CNMR spectrum of **3o**

**S.I. 31** ^1^H NMR spectrum of **3p**

**S.I. 32** ^13^ CNMR spectrum of **3p**

**S.I. 33** ^1^H NMR spectrum of **3q**

**S.I. 34** ^13^ CNMR spectrum of **3q**
